# 13-Methyltetradecanoic Acid Exhibits Anti-Tumor Activity on T-Cell Lymphomas *In Vitro* and *In Vivo* by Down-Regulating p-AKT and Activating Caspase-3

**DOI:** 10.1371/journal.pone.0065308

**Published:** 2013-06-07

**Authors:** Qingqing Cai, Huiqiang Huang, Dong Qian, Kailin Chen, Junhua Luo, Ying Tian, Tianxin Lin, Tongyu Lin

**Affiliations:** 1 Department of Medical Oncology, Cancer Center, Sun Yat-sen University, Guangzhou, P. R. China; 2 State Key Laboratory of Oncology in Southern China, Guangzhou, P. R. China; 3 Department of Urology, Sun Yat-sen Memorial Hospital, Sun Yat-sen University, Guangzhou, P. R. China; 4 Lin Bai-xin Medical Research Center, the Second Affiliated Hospital, Sun Yat-sen University, Guangzhou, P. R. China; Enzo Life Sciences, Inc., United States of America

## Abstract

13-Methyltetradecanoic acid (13-MTD), a saturated branched-chain fatty acid purified from soy fermentation products, induces apoptosis in human cancer cells. We investigated the inhibitory effects and mechanism of action of 13-MTD on T-cell non-Hodgkin’s lymphoma (T-NHL) cell lines both *in vitro* and *in vivo*. Growth inhibition in response to 13-MTD was evaluated by the cell counting kit-8 (CCK-8) assay in three T-NHL cell lines (Jurkat, Hut78, EL4 cells). Flow cytometry analyses were used to monitor the cell cycle and apoptosis. Proteins involved in 13-MTD-induced apoptosis were examined in Jurkat cells by western blotting. We found that 13-MTD inhibited proliferation and induced the apoptosis of T-NHL cell lines. 13-MTD treatment also induced a concentration-dependent arrest of Jurkat cells in the G_1_-phase. During 13-MTD-induced apoptosis in Jurkat cells, the cleavage of caspase-3 and poly ADP-ribose polymerase (PARP, a caspase enzymolysis product) were detected after incubation for 2 h, and increased after extending the incubation time. However, there was no change in the expression of Bcl-2 or c-myc proteins. The appearance of apoptotic Jurkat cells was accompanied by the inhibition of AKT and nuclear factor-kappa B (NF-κB) phosphorylation. In addition, 13-MTD could also effectively inhibit the growth of T-NHL tumors *in vivo* in a xenograft model. The tumor inhibition rate in the experimental group was 40%. These data indicate that 13-MTD inhibits proliferation and induces apoptosis through the down-regulation of AKT phosphorylation followed by caspase activation, which may provide a new approach for treating T-cell lymphomas.

## Introduction

T-cell lymphomas are a heterogeneous group of non-Hodgkin’s lymphomas (NHLs), whose incidence accounts for 30% of non-Hodgkin’s lymphomas in Asia. T-cell lymphomas are more aggressive and prognosis is poorer compared with that of B-cell lymphomas [1]–[3]. So far, there is no standard treatment strategy for T-cell non-Hodgkin’s lymphomas (T-NHLs). Saturated and unsaturated fatty acids have been reported to have antineoplastic effects [4]–[7]. 13-Methyltetradecanoic acid (13-MTD), a saturated branched-chain fatty acid purified from soy fermentation products, can inhibit the growth of various cancer cell lines (e.g. breast cancer cells, prostate cancer cells, hepatocellular carcinoma cells, leukemia cells, human bladder cancer cells) *in vitro* or *in vivo* (human hepatocellular carcinoma LCI-D35 and human prostate cancer DU 145 cell lines) by inducing apoptosis without significant toxic side effects [8], [9], [10]. The median lethal dose (LD_50_) for 13-MTD suggested that mice could sustain oral feeding of 5 g/kg/day without observable adverse events [8]. 13-MTD administered orally is absorbed by the intestine and transported primarily as chylomicrons in the lymphatic system and then into the circulation through the thoracic duct. Thus, the concentration of drug will be relatively high in the lymphatic system. Therefore, we predicted that 13-MTD, a broad-spectrum high-performance drug, would be useful for treating NHL, especially T-NHL, which is less responsive to standard chemotherapy regimens [11]. The resistance of T-cell lymphomas to chemotherapeutic agents is quite complex. One of the reasons for resistance to chemotherapeutic agents may be linked to the presence of multidrug resistance (MDR) proteins and the activation of some oncogenes or oncogenic factors (e.g., Bcl-2, Bcl-xl, AKT, NF-κB, ras or mutant P53) are also considered as underlying mechanisms [12]–[16].

Abnormal apoptosis is associated with the initiation and development of malignant tumors. The serine/threonine kinase AKT plays a central role in tumorigenesis. The biological significance of AKT kinase activity in lymphomagenesis has been established in a mouse model [9]. Furthermore, high phospho (p)-AKT expression is associated with short survival in diffuse large B-cell lymphoma (DLBCL) cell lines [17]–[19], whereas overexpression of AKT can inhibit apoptosis [20], [21]. The phosphorylation of AKT may alter the activity of proteins such as caspase-3, Bcl-2 family members, nuclear factor-kappa B (NF-κB) and other transcription factors that induce or inhibit apoptosis [19].

Therefore, we speculated that 13-MTD might induce apoptosis in T-NHL cells by down-regulating p-AKT, which is important for NHL cell survival. In the present study, we investigated the anti-tumor effect of 13-MTD on T-NHL cell lines *in vitro* and *in vivo*, and examined the involvement of AKT and its downstream signaling pathway to elucidate the possible cytostatic mechanism of 13-MTD on T-NHL cells.

## Results

### 13-MTD Inhibits T-NHL Cell Growth

**Figure 1:**
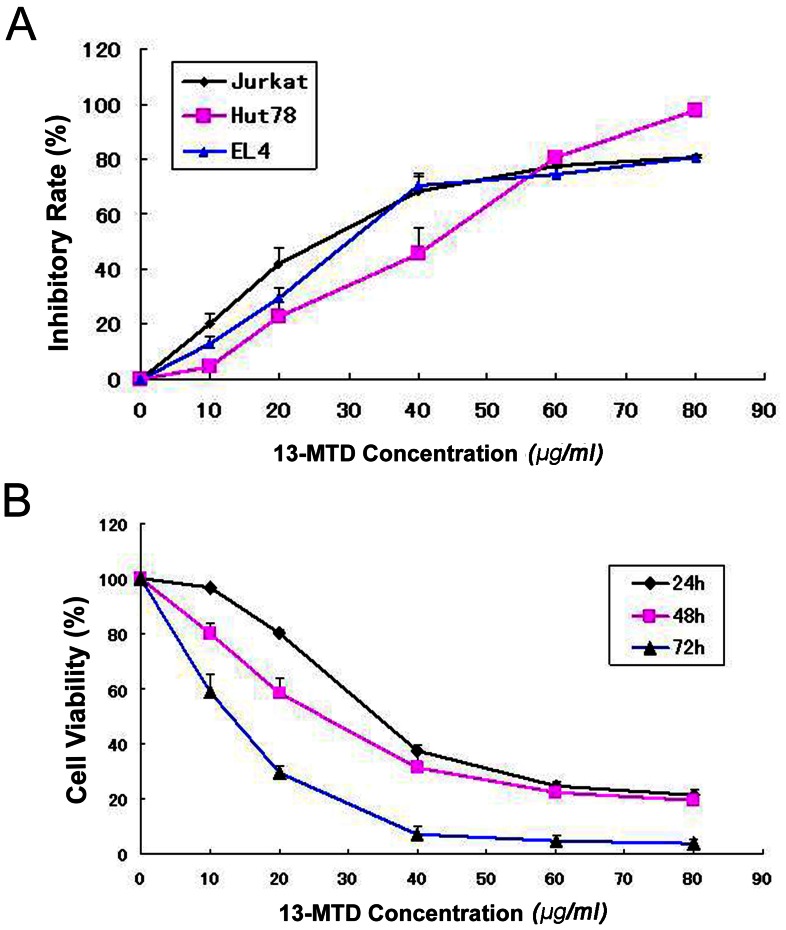
Inhibition of proliferation of Jurkat, Hut78 and EL4 cells by 13-MTD treatment. (**A**) Cultivation with 13-MTD for 48 hours at different concentrations (10, 20, 40, 60, 80 µg/ml) inhibited the proliferation of Jurkat, Hut78 and EL4 cells in a dose-dependent manner. (**B**) Cell viability of 13-MTD-treated Jurkat cells decreased in a time-dependent manner at different incubation time points (24, 48, 72 h).

The growth inhibitory effect of 13-MTD on T-NHL cells *in vitro* was determined by the cell counting kit-8 (CCK-8) assay. 13-MTD had a potent anticancer activity on T-NHL cell lines. After incubation of Jurkat cells, Hut78 cells and EL4 cells with various concentrations of 13-MTD for 48 h, the number of T-NHL cells was reduced dramatically in a dose-dependent manner (Figure 1A). The half-maximal inhibitory concentration (IC_50_) values of 13-MTD at 48 h were determined for the following cell lines: Jurkat cells, 25.74±3.50 µg/ml; Hut78 cells, 31.29±2.27 µg/ml; and EL4 cells, 31.53±5.18 µg/ml. The antiproliferative effects of 13-MTD on Jurkat cells were measured at different time points (Figure 1B). The inhibitory effects of 13-MTD on Jurkat cells were enhanced with increasing incubation time. The IC_50_ values of 13-MTD at 24 h, 48 h and 72 h were as follows: 38.51±0.72 µg/ml; 25.74±3.50 µg/ml; and 11.82±0.90 µg/ml, respectively. These data suggest that 13-MTD inhibits the proliferation of T-NHL cells in a dose- and time-dependent manner.

### 13-MTD Induces G_1_-phase Arrest of T-NHL Cells

**Figure 2:**
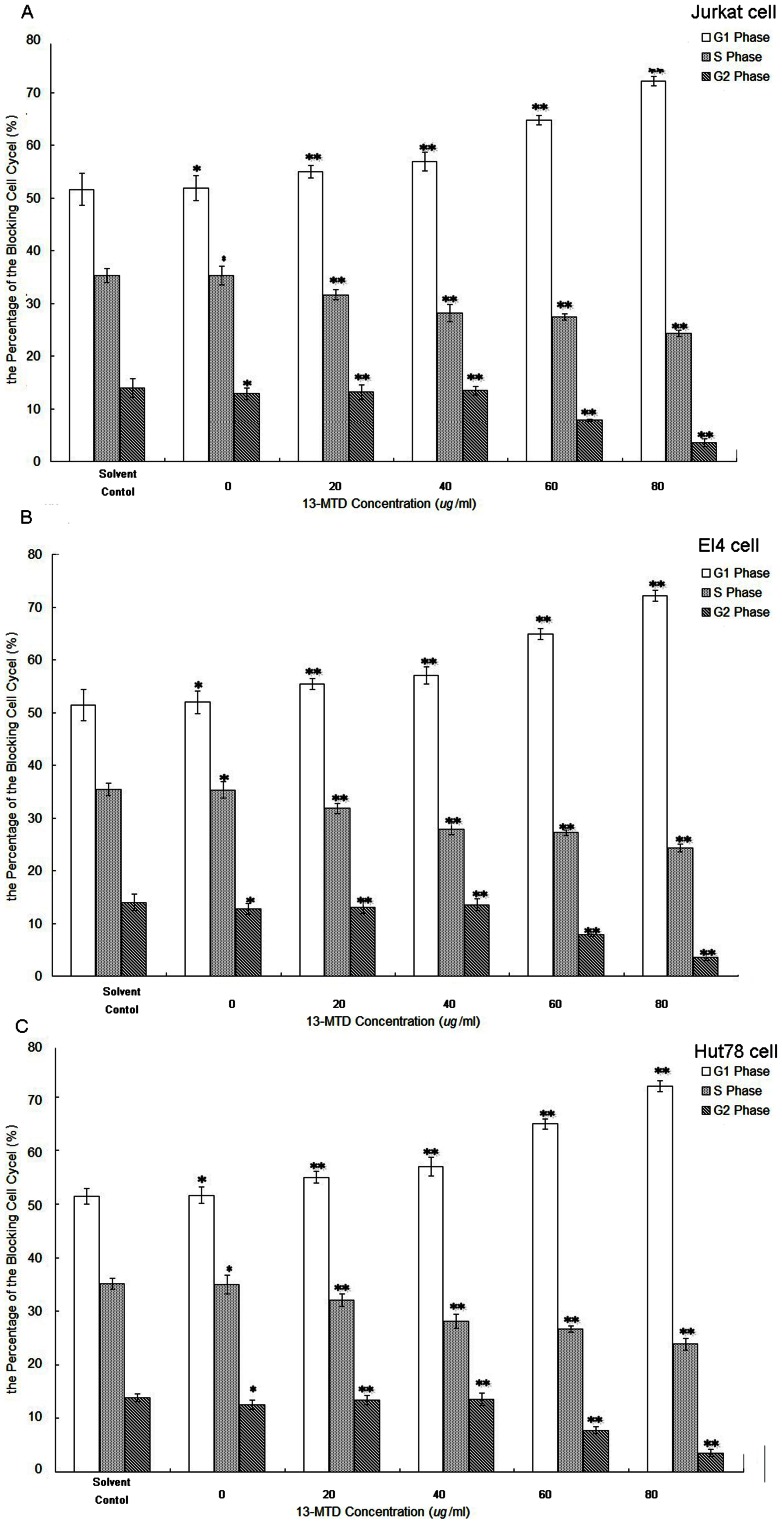
The effects of 13-MTD in the G_1_ phase arrest in T-NHL cells. Cells at a density of 2 × 10^6^ cells per well were treated with 13-MTD at different concentrations (0, 20, 40, 60, 80 µg/ml) for 48 h and harvested to evaluate the cell cycle distribution. The percentage of cells in G_1_, S, and G_2_ phases in Jurkat (**A**), EL4 (**B**) and Hut78 (**C**) cells are shown. *P>0.05, ** *P*<0.05 compared with the solvent group. All data are derived from three individual experiments with triplicate wells.

To better understand the effect of 13-MTD on the growth of T-NHL cells, we performed flow-cytometric analysis to determine the cell cycle distribution. Cultivation of Jurkat cells with various concentrations of 13-MTD for 48 h caused G_1_ arrest. As shown in Figure 2A, the percentage of G_1_ phase cells significantly increased in Jurkat cells treated with 13-MTD compared with solvent treatment (*P*<0.01), whereas the percentage of Jurkat cells in S and G_2_ phase decreased gradually (*P*<0.01). These results demonstrated that 13-MTD exerted its effects on Jurkat cells by G_1_ phase cell cycle arrest rather than S phase and G_2_ phase arrest, which possibly contributed to the decreased viability observed. Similar results were obtained in EL4 and Hut78 cell lines (Figure 2B and 2C, respectively).

### 13-MTD Induces Apoptosis in T-NHL Cells

**Figure 3:**
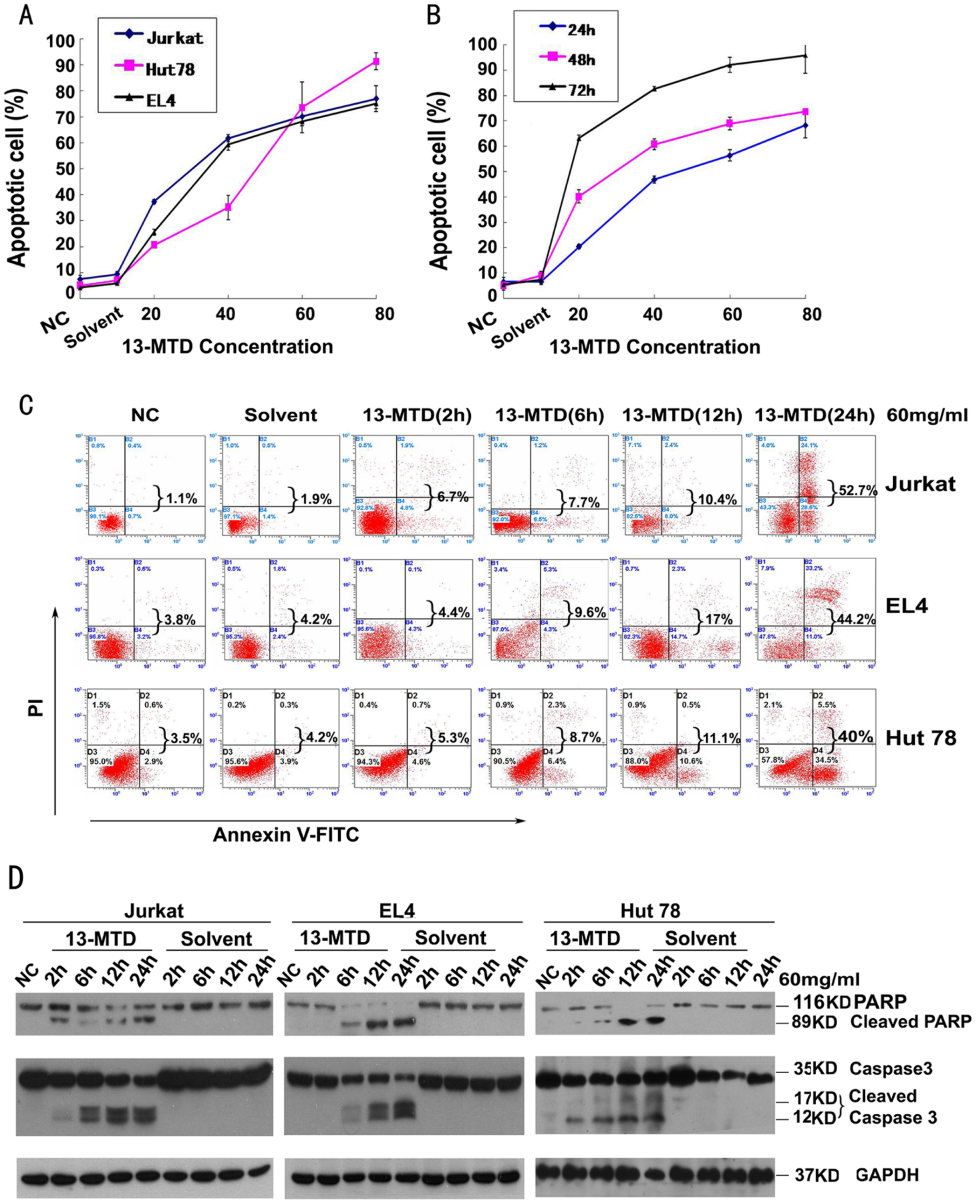
13-MTD induces apoptosis, pro-caspase-3 and PARP cleavage in T-NHL cells. Apoptotic cells were detected by flow cytometry after Annexin V/PI staining, and caspase-3 and PARP were detected by Western blot analysis. (NC, negative control) (**A**) 13-MTD induced apoptosis in Jurkat, Hut78, and EL4 cells after 48 h of 13-MTD treatment at different concentrations (0, 20, 40, 60, 80 µg/ml). The number of apoptotic cells increased in a dose-dependent manner. Values represent the means ± standard deviation (SD) of three independent experiments. (**B**) 13-MTD induced apoptosis in Jurkat cells at different incubation time points (24, 48, 72 h). Values represent the means ± SD of three independent experiments. (**C**) Jurkat, EL4 and Hut78 cells were incubated with 13-MTD or solvent control at 60 µg/ml for the indicated time periods. Each sample was stained with Annexin V/PI and analyzed by flow cytometry. The ratio of cells is shown in each quadrant. The percentage written behind the braces refers to the ratio of apoptosis cells in each sample. Data shown are representative of three independent experiments. (**D**) Jurkat,EL4 and Hut78 cells were treated with 60 µg/ml 13-MTD or solvent control for 2, 6, 12 and 24 h. Cells were then collected, lysed and subjected to western blot analysis with PARP and caspase-3 antibodies that can detect cleaved PARP and cleaved caspase-3. GAPDH was used as a loading control. All data are derived from three individual experiments with triplicate wells.

Flow-cytometric analysis demonstrated that 13-MTD caused a time- and dose-dependent increase in T-NHL cell apoptosis (Figure 3A, 3B, and Table 1). Apoptosis of Jurkat cells significantly increased after 12 h treatment compared with solvent treatment groups, and further increased when treated with 20–80 µg/ml 13-MTD over 24–48 h (*P*<0.05). The number of apoptotic cells at 48 h following 20–80 µg/ml 13-MTD treatment increased significantly compared with the solvent treatment groups (*P*<0.05). There was no difference in apoptosis of T-NHL cells between the untreated control groups and the corresponding solvent group. Similar results were observed in the other two cell lines. These results demonstrated the ability of 13-MTD to induce apoptosis in T-NHL cells.

**Table 1:** 13-MTD induced apoptosis in Jurkat, Hut78 and EL4 cells.

13-MTD (µg/ml)	Incubation time				
	12 h	24 h	48 h		
	Jurkat cells	Jurkat cells	Jurkat cells	Hut78 cells	EL4 cells
solvent	6.60±0.36	8.93±1.69	8.33±1.04	3.27±0.81	3.90±0.79
0	6.60±0.36***	8.93±1.69*	7.43±1.59***	5.00±1.04***	4.17±0.45*
20	5.43±1.10**	8.23±2.65**	23.33±0.85**	5.60±0.17**	5.60±1.15**
40	8.93±0.86**	10.80±2.18**	42.77±1.36**	21.10±4.75**	52.30±2.13**
60	13.43±3.01**	19.00±2.46**	85.17±2.25**	83.67±9.77**	72.20±1.51**
80	48.37±7.18**	63.70±1.27**	89.00±4.98**	91.43±3.29**	90.17±2.10**

13-MTD: 13-Methyltetradecanoic acid. Each value represents the mean ± SD of three independent experiments.

* *P*>0.05,

** *P*<0.01,

*** *P*>0.01 compared with the solvent control group.

### 13-MTD Induces Pro-caspase-3 and PARP Cleavage, and Inhibits AKT and NF-κB Phosphorylation

**Figure 4:**
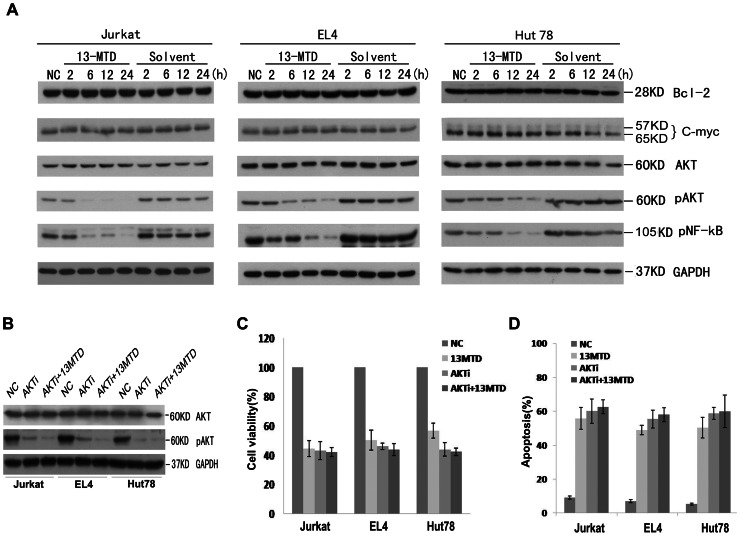
Western blot analysis of the expression of Bcl-2, c-myc, AKT, p-Akt, p-NF-κB in Jurkat EL4 and Hut78 cells treated with 60 µg/ml solvent control or 13-MTD for 2, 6, 12, 24 h. **GAPDH was used as a loading control.** There was no change in the expression of Bcl-2, c-myc or AKT proteins. The expression of phosphorylated AKT and NF-κB were decreased in a time-dependent manner with 13-MTD treatment. All data are derived from three individual experiments with triplicate wells. (B) These three cell lines were treated with solvent or 13-MTD (60 mg/ml) for 24 hours, followed by Akt inhibitor V (40 uM) exposure for 30 min. The phosphorylation of AKT was significantly inhibited by Akt inhibitor V. The anti-growth (C) and apoptosis-promoting (D) effects of 13-MTD on T-NHL cells almost disappeared once the phosphorylation of AKT was inhibited. Result are representative of there independent experiments. All the values represent means ±standard deviation (S.D.).

To explore the molecular mechanism of apoptosis induced by 13-MTD, we studied the generation of specific biochemical apoptotic markers after exposure of Jurkat, Hut78 and EL4 cells to 60 µg/ml 13-MTD for 2, 6, 12 or 24 h. Pro-caspase-3 was cleaved into its active form after 13-MTD treatment for 2 h in Jurkat cells and 6 h in Hut78 and EL4 cells, and then increased in a time-dependent manner along with the apoptotic cells, as detected by flow cytometric analysis (Figure 3C, 3D). The cleavage of its downstream substrate poly ADP-ribose polymerase (PARP, 85-kDa band) was also observed after 2 h in Jurkat cells and 6 h in Hut 78 and EL4 cells incubated with 13-MTD, and increased in a time-dependent manner (Figure 3D). However, there was no observed change in the expression of Bcl-2 or c-myc proteins compared with the control group after 13-MTD treatment for 24 h (Figure 4). Interestingly, the level of AKT phosphorylation was significantly decreased after 13-MTD treatment compared with the vehicle-treated cells (*P*<0.05; Figure 4). The time point of AKT phosphorylation correlated with the appearance of apoptosis. Moreover, NF-κB phosphorylation was inhibited after incubation with 13-MTD for 12 and 24 h. The down-regulation of NF-κB phosphorylation was observed after the down-regulation of AKT phosphorylation. Thus, 13-MTD suppressed AKT and NF-κB activation, and induced apoptosis of T-NHL cells by activation of the caspase pathway. Additionally, the apoptotic effect of 13-MTD disappeared almost completely after the cellular phosphorylation of AKT was inhibited by AKT inhibitor V in the three cell lines (Figure 4B). These results further prove that AKT phosphorylation is directly involved in the mode of action of 13-MTD.

### 13-MTD Administration Suppresses Tumor Growth in Nude Mice

**Figure 5:**
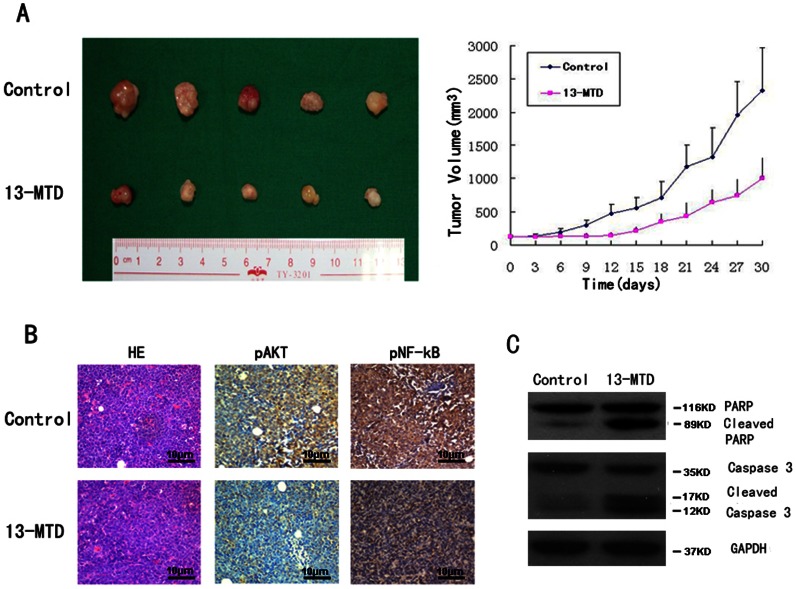
The therapeutic effect of 13-MTD on Jurkat cell xenografts. The tumor volumes of xenografts were measured with calipers every 3 days for a total of 30 days after the start of treatment. After 30 days of treatment, mice were sacrificed and the tumors removed and photographed. (**A**) Tumor growth was significantly suppressed with 13-MTD treatment. The respective tumor volumes from the solvent control and 13-MTD treatment groups were 2325.43±318.32 mm^3^ and 1000.54±156.78 mm^3^ (n = 5, *P*<0.01, Student’s *t*-test) (**B**) Tumors were then fixed and stained with H&E to examine tumor cell morphology. IHC showed decreased phosphorylation of AKT and NF-κB in the tumor tissue after treatment with 13-MTD. (Original magnification×100) (**C**) 13-MTD enhanced the activation of caspase-3 and PARP proteins in tumor xenografts compared with controls. Tumor lysates were subjected to the analysis of protein levels using western blot analysis. GAPDH was used as a loading control. Representative blots are shown from independent experiments with six different tumors in each treatment group.

BALB/c nude mice were injected subcutaneously with Jurkat lymphoma cells (5 × 10^6^ cells per mouse in a 0.2-ml volume). There was no significant difference between groups in weight and tumor volume of mice before 13-MTD treatment. After 5 days of 13-MTD treatment (70 mg/kg/day orally), the tumor volume was smaller in the treatment group compared with control groups (Figure 5A). The mean tumor weight in nude mice receiving 13-MTD treatment was significantly lower than in the control group (1.32±0.32 g vs 2.68±0.76 g, *P*<0.05). Furthermore, the results of immunohistochemistry (IHC) and western blot analysis showed that p-AKT, and p-NF-kB were decreased and the cleavage of pro-caspase-3 and PARP was significantly increased in the xenograft tumor tissue, consistent with the *in vitro* results (Figure 5B and 5C). However, there was no significant difference between the mean body weight of nude mice in each group after treatment (18.29±1.50 vs 18.14±1.35 g; *P*>0.5). Hematoxylin & eosin (H&E) staining of the internal organs of mice showed no obvious pathological changes during the experiment (Figure S1). A similar effect of 13-MTD was demonstrated in the nude mouse xenograft model using EL4 cells (Table S1, Figure S2). Therefore, 13-MTD can effectively inhibit the growth of T-NHL tumors in nude mice.

## Discussion

13-MTD is the most abundant component of a soy fermentation product, and its anti-cancer properties have been observed in many cell lines isolated from breast cancer, prostate cancer, hepatocellular carcinoma, leukemia and bladder cancer [8], [9], [20]. In this report, we studied the effects of 13-MTD on T–NHL *in vitro* and *in vivo*. Our results demonstrated that 13-MTD could inhibit proliferation and induce apoptosis in different T-NHL cells in a dose- and time-dependent manner. Additionally, 13-MTD could effectively inhibit the growth of T-cell NHL *in vivo* in a xenograft model. Furthermore, flow cytometric analysis showed that cell cycle arrest occurred at the G_1_ phase in Jurkat, Hut78 and EL4 cells after incubation with 13-MTD in a concentration-dependent manner, resulting in cell cycle dysfunction and eventually leading to apoptosis.

Caspases, a family of intracellular cysteine proteases, are the effector components of apoptosis and are initially produced as inactive zymogens (procaspases). Caspases are divided into two functional subfamilies: initiator caspases (caspase-8, -9, -10) that are involved in regulatory events, and effector caspases (caspase-3, -6, -7) that are responsible for cell disassembly events. Activation of caspase-3 is a key event in initiating programmed cell death [22], [23]. PARP, a 116-kDa nuclear chromatin-associated protein, is also involved in many genomic processes such as apoptosis. During apoptosis, PARP is the specific cellular substrate for caspase-3/7 and is cleaved into 89- and 24-kDa fragments that contain the active site and the DNA-binding domain of the enzyme, respectively [24]–[26].

Because 13-MTD induced apoptosis in T-NHL cells, we further investigated the cellular mechanisms by which this might happen. We detected the expression of caspase-3 proteins in 13-MTD-treated (60 µg/ml) Jurkat, Hut78 and EL4 cells using western blot analysis. The activity of caspase-3 was initiated after 2 and 6 h of 13-MTD treatment in Jurkat cells, and Hut78 and EL4 cells, respectively. The inactive pro-enzyme was activated and converted into active fragments, which increased in a time-dependent manner. Consistent with caspase-3 activation, 13-MTD induced the proteolytic cleavage of PARP as measured by the accumulation of 89-kDa fragments in Jurkat, Hut 78 and EL4 cells. The apoptotic fragments increased after 6 h treatment in Jurkat cells and 12 h for Hut 78 and EL4 cells. This suggests that 13-MTD can induce apoptosis in Jurkat, Hut 78 and EL4 cells mediated by caspase-3.

Bcl-2 family proteins play a key role in the occurrence and regulation of apoptosis, but also in the sensitivity of NHL to chemotherapy [27], [28]. C-myc protein, activated by cytokines, induces cells to leave the G_0_ cell cycle phase and enter the S phase. High levels of Bcl-2 and c-myc protein expression are associated with poor prognosis in NHL patients [29]. However, in the present study, there was no change in the expression of Bcl-2 and c-myc proteins, contrary to previous reports in bladder cancer cells [10]. Questions remain concerning whether changes in the levels of other Bcl-2 family proteins will change the drug effects of 13-MTD in treated cells, and will be an important area for future studies. Additional mechanisms of 13-MTD-induced apoptosis may also exist in T-NHL cells.

AKT, a serine–threonine kinase, is intimately involved in the regulation of cell survival [30], and is activated by a number of growth factor signaling pathways [31]. Activated AKT can regulate downstream target proteins, including glycogen synthase kinase (GSK), caspase-9, Bad and NF-κB, to promote proliferation, angiogenesis and anti-apoptosis of cells during chemotherapy or radiotherapy [16], [32]–[34]. Previously it was shown that overexpression and dysfunction of AKT phosphorylation was common in human cancers, such as ovarian cancer, breast cancer, prostate cancer, lung cancer and malignant lymphoma [8], [10]. Our recent study demonstrated that p-AKT expression is an independent prognostic factor for peripheral T-cell lymphoma [14]. Furthermore, we observed that down-regulation of AKT phosphorylation after 2 h (Jurkat cells) or 6 h (Hut78 and EL4 cells) treatment with 13-MTD correlated with the appearance of apoptosis. The killing effect of 13-MTD was almost disappeared after the cellular phosphorylation of AKT being inhibited by AKT inhibitor V in these three cell lines. This suggests that AKT phosphorylation may be crucial for 13-MTD-induced apoptosis, consistent with previous reports regarding bladder cancer cells [10] and DLBCL cell lines [19].

The up-regulation of AKT may activate the downstream anti-apoptosis cascade reaction. Barancík *et al* [35] observed that LY294002, which inhibited AKT activation and other downstream targets, promoted apoptosis of L1210/VCR, a murine leukemia cell line with multidrug resistance. Additionally, inhibition of AKT affected the formation of NHL multidrug resistance. However, down-regulation of p-AKT may sensitize drug-resistant lymphoma cells [34], [36], [37]. These results suggest that the sustained activation of AKT is an important factor in the development and resistance of NHL, and thus is a potential therapeutic target [38]. Recently, antitumor drugs targeting AKT have been tested at a pre-clinical level to determine the potential for severe adverse events or poor selectivity [39], [40]. Therefore, as 13-MTD also targets the phosphorylation of AKT with high efficacy and low toxicity, it may be a potential candidate for the treatment of NHL.

NF-κB is an important intracellular nuclear transcription factor, as it binds to a variety of gene promoters and enhances gene transcription and expression related to immunization, inflammatory and stress-related events, and the regulation of cell proliferation and apoptosis. AKT is a key regulator of NF-κB-dependent gene transcription. In this study, NF-κB phosphorylation was significantly decreased after 12–24 h treatment with 13-MTD compared with the control group, indicating that changes in p-NF-κB might be related to apoptosis. However, the decrease of p-NF-κB was observed after the change in p-AKT [41]. As AKT activation promotes the transcription of NF-κB, the relevance of decreased levels of p-NF-κB needs to be elucidated.

In summary, 13-MTD is a saturated branched-chain fatty acid purified from soy fermentation products. Our results demonstrated that 13-MTD could effectively inhibit T-NHL growth *in vitro* and *in vivo* by inhibiting proliferation and inducing apoptosis though AKT phosphorylation followed by caspase activation. 13-MTD thus presents a potential chemotherapy agent for the treatment of T-NHL.

## Materials and Methods

### Ethics Statement

This study was carried out in strict accordance with the recommendations in the Guide for the Care and Use of Laboratory Animals of the National Institutes of Health. All animal experiments were approved by the Committee on the Ethics of Animal Experiments of Cancer Center, Sun Yat-sen University. (Permit Number: 2008-0002). All surgery was performed under sodium pentobarbital anesthesia, and all efforts were made to minimize suffering.

### Materials

13-MTD was purchased from Sigma (St. Louis, MO, USA). Annexin V/PI detection kits were purchased from Bender (Vienna, Austria). Mouse monoclonal antibodies specific for caspase-3, PARP, c-myc and Bcl-2 were purchased from Santa Cruz (Santa Cruz, CA, USA). Mouse monoclonal antibodies specific for GAPDH and β-actin, and rabbit monoclonal antibodies specific for p-AKT, AKT and p-NF-κB p65 were purchased from Cell Signal (Danvers, MA, USA). The mouse monoclonal antibody specific for anti-c-myc was from BD Bioscience (Sparks, MD, USA). Akt Inhibitor V, Triciribine, was purchased from Merck (Darmstadt, Germany).

### Cell Lines

The T-NHL cell lines, Jurkat cells, Hut78 cells, and EL4 cells were obtained from American Type Culture Collection (Manassas, VA, USA). Jurkat and Hut78 cells were cultured with RPMI 1640 Media (Gibco Laboratories, Buffalo, Grand Island, NY, USA), and EL4 cells were maintained in DMEM (Gibco Laboratories). All cultures were supplemented with 10% (vol/vol) fetal bovine serum (Gibco Laboratories), 100 units/ml potassium penicillin G (Huabei Chemical Co., Shijiazhuang, China) and 100 units/ml streptomycin sulfate (Huabei Chemical Co.) at 37°C in a 5% CO_2_ incubator.

### CCK-8 Assay

Cell proliferation was estimated using a CCK-8 assay [42]. Jurkat, Hut78 and EL4 cells were used for the logarithmic growth phase. Cell suspensions (8000 cells/well) were added to 96-well plates in a volume of 200 µl/well. The treatment group, solvent control group and control group were treated with different doses of 13-MTD, the corresponding solvent (Tween 80), culture media and CCK-8, respectively. The final concentrations of 13-MTD were 80, 60, 40, 20, 10 µg/ml, respectively. Each group was prepared with five parallel wells and incubated at 37°C, 5% CO_2_, for 48 h. In addition, Jurkat, Hut78 and EL4 cells were exposure to Akt inhibitor V (40 uM) for 30 min after treatment with solvent or 13-MTD (60 mg/ml) for 24 hours. At the end of the culture period, 10 µl CCK-8 was added to each well. After 4 h incubation, the absorbance was measured with an enzyme calibrator at 450 nm and 630 nm and the optical density (OD) values were measured. Inhibition of cell growth was calculated using the following formula: percentage of inhibition = (ODA-ODT)/(ODA-ODC) × 100%, where ODT, ODC and ODA are the OD values of the treated sample, the control sample and the solvent control sample, respectively. The IC_50_ value was calculated using the Bliss method. The effects on proliferation of 13-MTD in Jurkat, Hut78 and EL4 cells over 24 h and 48 h were also evaluated by the above method. Experiments were repeated three times.

### Flow Cytometric Analysis of Cell Cycle Distribution

After 48 h incubation with different doses of 13-MTD or the solvent control, T-NHL cells (Jurkat,Hut78 and EL4 cells) were washed twice with phosphate-buffered saline (PBS), and fixed in 70% ethanol overnight at 4°C. The fixed cells were pelleted by centrifugation at × 2000 r/min for 10 min, re-suspended in PBS, stained with 10 mg/ml propidium iodide (PI) (Sigma Co., St.Louis, MO) for 5 min, and incubated in the dark at 4°C for 30 min as previously described [43].The DNA content of cells was analyzed using a model 100 flow cytometer (Beckman Coulter Inc., Kraeme, CA, USA) with excitation and emission settings of 488 nm and 610 nm, respectively. The proportion of sub-G_1_ cells of each sample was detected according to the relative DNA content measured by the flow cytometer, using LYSIS software (Becton Dickinson Co.).

### Flow Cytometric Analysis of Apoptosis

Cell suspensions were plated at a density of 2 × 10^6^ cells per 6-cm dish and treated with different doses of 13-MTD or the solvent control. Jurkat, Hut78 and EL4 cells were incubated for 2, 6, 12, 24 or 48 h. Or these three cell lines were treated with solvent or 13-MTD (60 mg/ml) for 24 hours, followed by Akt inhibitor V (40 uM) exposure for 30 min. The cells were pelleted by centrifugation at × 1500 r/min for 5 min and washed twice with PBS. After centrifugation at 1000×*g* for 5 min, cells were incubated with annexin V fluorescein isothiocyanate (FITC) for 10 min at room temperature (20–25°C) in the dark. Thereafter, cells were resuspended in Annexin V-FITC (190 µl) and 10 µl PI was added according to the protocol of the Annexin V/PI kit. The samples were then incubated in the dark and subjected to flow cytometry evaluation.

### Western Blot Analysis

Briefly, cells were harvested, washed, incubated with 13-MTD for 2, 6, 12 or 24 h or were incubated with Akt inhibitor V (40 uM) for 30 min after solvent or 13-MTD (60 mg/ml) treatment for 24 hours, and then lysed with SDS buffer (50 mmol/l Tris-HCl, 2% SDS, 0.1% bromophenol blue, 10% glycerine, 100 mmol/l DTT). Protein concentration in the resulting lysate was determined using ultraviolet spectrophotometry. Proteins in the supernatant were separated by 10 or 12% sodium dodecyl sulfate-polyacrylamide gel electrophoresis (SDS–PAGE) and transferred onto polyvinylidene fluoride (PVDF) (Roche Ltd, Basel, Switzerland) membranes as described previously [44]. Membranes were blocked with 5% nonfat dry milk overnight and incubated with the indicated primary antibody overnight. After washing, membranes were incubated with a secondary antibody. Finally, the membrane was washed, incubated with chemiluminescence reagents (Cell Signal Co.) and then exposed to X-ray film. As necessary, blots were stripped and re-probed with anti-GAPDH antibody as an internal control.

### Assessment of in vivo Tumor Growth

Two BALB/C nude mice were injected subcutaneously with Jurkat lymphoma cells (5 × 10^6^ cells per mouse in a 0.2 ml volume) into the right armpit. Another two mice were treated the same way using EL4 cells. The tumors were harvested after 7 days. The tumor tissues were cut into small pieces approximately 3 mm^3^. The fragments of Jurkat or EL4 lymphoma were each then implanted into twelve BALB/C nude mice [45], [46]. When the tumor size was about 5 mm (∼5 days after implantation), the mice were divided into two groups: the treatment group was treated with 13-MTD 70 mg/kg/day for 15 days orally by gavage in EL4 cell xenografts, and 30 days in Jurkat cell xenografts; the control group was administered the same volume of the solvent solution (Tween 80). After treatment, the tumor formation rate was calculated as [number of mice whose tumor size was greater than 5 mm]/[the total number of mice in the group]. Growth of subcutaneous tumors was measured using calipers. The greatest length of a tumor mass (a) and the width perpendicular to the length (b) were measured every 3 days, and tumor size was presented as 0.5 × ab^2^ [47]. The inhibition rate was calculated as [the weight of the treatment group]/[the weight of control group] × 100%, as previously described [8].

### H&E Staining Analysis and Immunohistochemistry

H&E staining analysis was performed as previously described [48]. IHC staining was performed on 5-µm tissue sections rehydrated through graded alcohols. Endogenous peroxidase activity was blocked with 0.3% hydrogen peroxide for 15 min. Antigen retrieval was performed by boiling sections in a microwave oven with 10 mM citrate buffer, pH 6.0, for 15 min. Nonspecific binding was blocked with 10% normal rabbit serum for 10 min. The tissue slides were incubated with the antibody overnight at 4°C. Subsequently, the slides were sequentially incubated with biotinylated goat anti-rabbit immunoglobulin (IgG) at a concentration of 1∶100 for 30 min at 37°C and then reacted with a streptavidin-peroxidase conjugate for 30 min at 37°C and 3′–3′ diaminobenzidine as a chromogen substrate. The nucleus was counterstained using Meyer’s hematoxylin. The negative control consisted of replacing the primary antibody with normal nonspecific IgG [49].

### Statistical Analysis

Data values were expressed as means ± SE. Student’s *t*-test was used for statistical analysis between the two treatment groups both *in vitro* and *in vivo*, and a *P* value <0.05 was considered statistically significant. A homogeneity test of variance was applied for the comparison between three treatment groups or more. If variance was homogeneous, a one-way analysis of variance was used and the LSD (least significant difference) method was employed for the comparison between any two groups with a significance level of *α* = 0.05. If variance was heterogeneous, a rank test was used and the Bonferroni method was applied for comparisons between any group and the group without 13-MTD-treatment with a significance level of *α* = 0.05. Statistical analysis was performed using the SPSS statistical software package (SPSS Standard version 13.0, SPSS Inc., Chicago, USA).

## Supporting Information

Figure S1 **H&E stain of mouse internal organs with or without 13-MTD treatment.** There were no significant pathological changes between the solvent control and 13-MTD treatment groups.
(TIF)

Figure S2 **The therapeutic effect of 13-MTD on EL4 cell xenografts.** The tumor volumes of xenografts were measured with calipers every 2 or 3 days for a total of 15 days after the start of treatment. **(A)** After 15 days of treatment, mice were sacrificed and the tumors were removed and photographed. The tumor volume of the 13-MTD group was significantly smaller (4697.76±1284.30 mm^3^) than in the solvent group (7420.88±1087.62 mm^3^) (n = 6, *P = *0.002, Student’s *t*-test). **(B)** The changes in tumor volume from nude mice after 13-MTD treatment compared with the solvent control group. After 5 days of 13-MTD treatment, the tumor volume was significantly smaller in the treatment group compared with the control groups (*P<0.05). Error bars are a graphical representation of the variability of data and are used on graphs to indicate the standard deviation.
(TIF)

Table S1 **Changes in tumor volume of naked mice after medication.**
(DOC)
