# Supplementary figures and images for: 13-Methyltetradecanoic Acid Exhibits Anti-Tumor Activity on T-Cell Lymphomas In Vitro and In Vivo by Down-Regulating p-AKT and Activating Caspase-3
Source: PLoS One. 2013 Jun 7;8(6):e65308. doi: 10.1371/journal.pone.0065308 (PMC3676434; doi:10.1371/journal.pone.0065308)

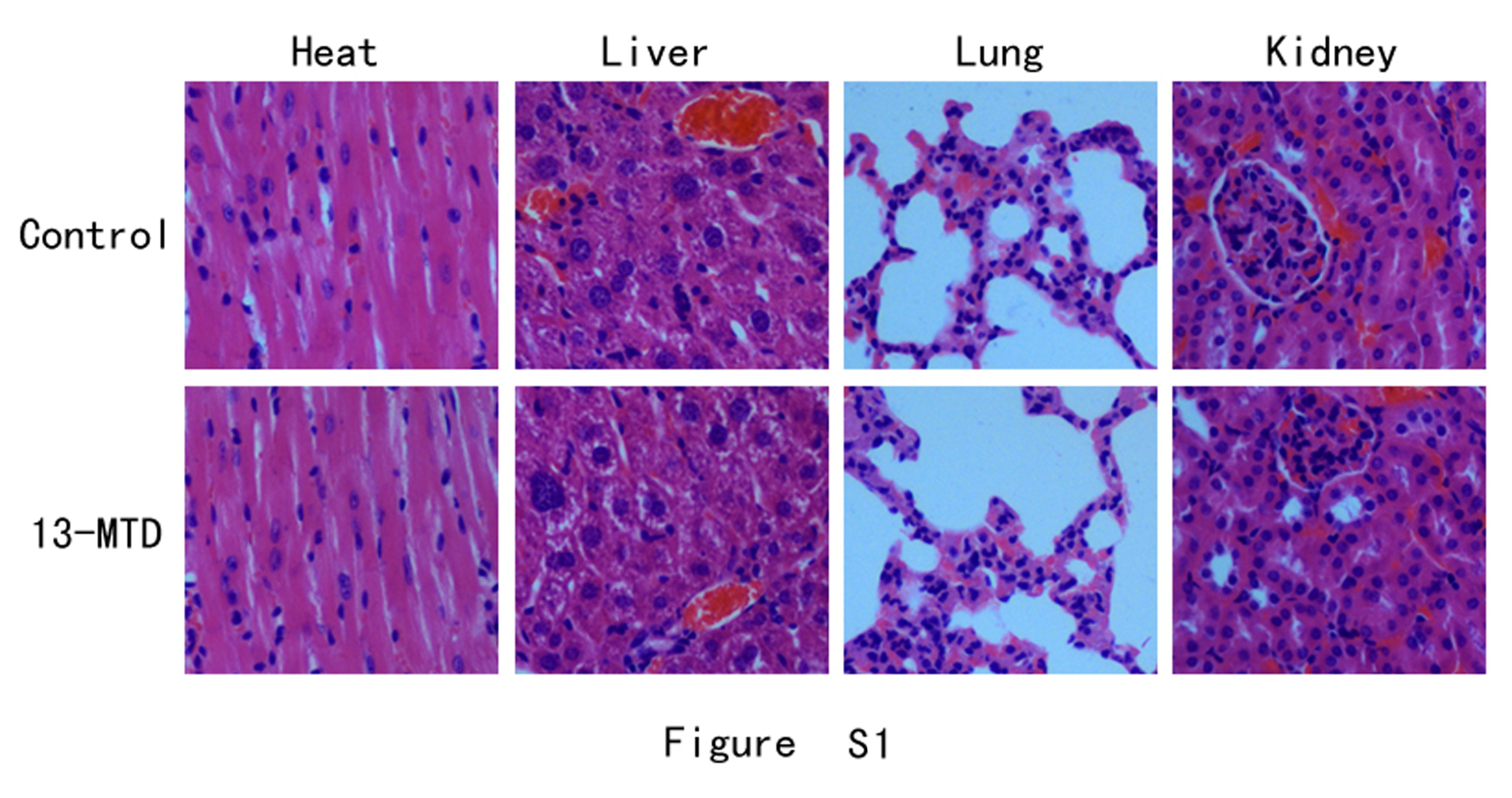

Supplement: Figure S1 — H&E stain of mouse internal organs with or without 13-MTD treatment. There were no significant pathological changes between the solvent control and 13-MTD treatment groups. (TIF) [file pone.0065308.s001.tif]

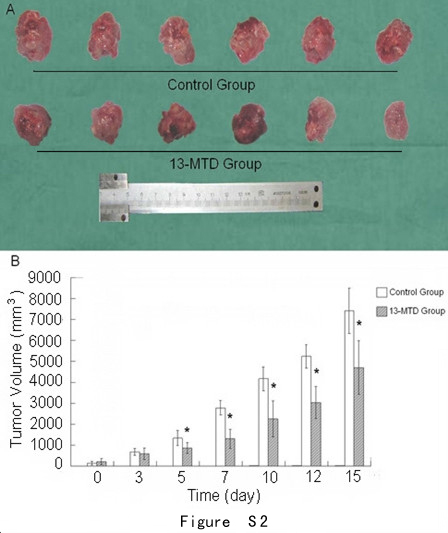

Supplement: Figure S2 — The therapeutic effect of 13-MTD on EL4 cell xenografts. The tumor volumes of xenografts were measured with calipers every 2 or 3 days for a total of 15 days after the start of treatment. (A) After 15 days of treatment, mice were sacrificed and the tumors were removed and photographed. The tumor volume of the 13-MTD group was significantly smaller (4697.76±1284.30 mm3) than in the solvent group (7420.88±1087.62 mm3) (n = 6, P = 0.002, Student’s t-test). (B) The changes in tumor volume from nude mice after 13-MTD treatment compared with the solvent control group. After 5 days of 13-MTD treatment, the tumor volume was significantly smaller in the treatment group compared with the control groups (*P<0.05). Error bars are a graphical representation of the variability of data and are used on graphs to indicate the standard deviation. (TIF) [file pone.0065308.s002.tif]
