# Supplementary material for: 13-Methyltetradecanoic Acid Exhibits Anti-Tumor Activity on T-Cell Lymphomas In Vitro and In Vivo by Down-Regulating p-AKT and Activating Caspase-3
Source: PLoS One. 2013 Jun 7;8(6):e65308. doi: 10.1371/journal.pone.0065308 (PMC3676434; doi:10.1371/journal.pone.0065308)
Supplement: Table S1 — Changes in tumor volume of naked mice after medication. (DOC) [file pone.0065308.s003.doc]

**Table S1.** Changes in tumor volume of naked mice after medication

| Time | Tumor volume（mm3）  tumor | |
| --- | --- | --- |
| （day） | Control | 13-MTD |
| d0 | 140.23± 87.04 | 209.30± 144.32 |
| d3 | 675.11±166.62 | 584.38± 274.19 |
| d5 | 1344.42±365.81 | 856.04± 253.90* |
| d7 | 2781.26±341.56 | 1303.74± 453.38* |
| d10 | 4171.60±563.44 | 2253.99± 866.82* |
| d12  1212 | 5241.96±560.62 | 3036.60± 767.64* |
| d15 | 7420.88±1087.62 | 4697.76±1284.30* |

13-MTD: 13-Methyltetradecanoic acid.

**P* <0.05 compared with the control group
